# Identification of QTL Associated with Regrowth Vigor Using the Nested Association Mapping Population in Switchgrass

**DOI:** 10.3390/plants11040566

**Published:** 2022-02-21

**Authors:** Santosh Nayak, Hem Bhandari, Malay C. Saha, Shahjahan Ali, Carl Sams, Vince Pantalone

**Affiliations:** 1 Department of Plant Sciences, The University of Tennessee, 2505 E J Chapman Drive, Knoxville, TN 37996, USA; hemnmsu@gmail.com (H.B.); carlsams@utk.edu (C.S.); vpantalo@utk.edu (V.P.); 2 Noble Research Institute, 2510 Sam Noble Parkway, Ardmore, OK 73401, USA; msaha06@yahoo.com (M.C.S.); shahjahan.ali8@gmail.com (S.A.)

**Keywords:** switchgrass (*Panicum virgatum* L.), nested association mapping (NAM) population, quantitative trait loci (QTL), single nucleotide polymorphism (SNP), regrowth vigor

## Abstract

Switchgrass (*Panicum virgatum* L.) is a warm-season perennial grass species that is utilized as forage for livestock and biofuel feedstock. The stability of biomass yield and regrowth vigor under changing harvest frequency would help manage potential fluctuations in the feedstock market and would provide a continuous supply of quality forage for livestock. This study was conducted to (i) assess the genetic variation and (ii) identify the quantitative trait loci (QTL) associated with regrowth vigor after multiple cuttings in lowland switchgrass. A nested association mapping (NAM) population comprising 2000 pseudo F_2_ progenies was genotyped with single nucleotide polymorphism (SNP) markers derived from exome-capture sequencing and was evaluated for regrowth vigor in 2017 and 2018. The results showed significant variation among the NAM families in terms of regrowth vigor (*p* < 0.05). A total of 10 QTL were detected on 6 chromosomes: 1B, 5A, 5B, 6B, 7B, and 8A, explaining the phenotypic variation by up to 4.7%. The additive genetic effects of an individual QTL ranged from −0.13 to 0.26. No single QTL showed a markedly large effect, suggesting complex genetics underlying regrowth vigor in switchgrass. The homologs of candidate genes that play a variety of roles in developmental processes, including plant hormonal signal transduction, nucleotide biosynthesis, secondary metabolism, senescence, and responses to both biotic and abiotic stresses, were identified in the vicinity of QTL.

## 1. Introduction

Switchgrass (*Panicum virgatum* L.) is a warm-season, perennial grass that is recognized as an important component of North American tallgrass prairies [1]. The natural population of switchgrass is reported to exist across a broad range of environments. Based on the natural habitat, switchgrass cultivars are mainly classified into two distinct ecotypes: lowland and upland [2,3]. Lowland ecotypes are characterized as having taller plants, thicker stems, fewer tillers, and are adapted to relatively warm and wet environments compared to upland ecotypes [1]. Switchgrass ecotypes comprise varying ploidy levels. Lowland ecotypes are mostly tetraploids (2n = 4x = 36), whereas upland ecotypes are predominantly octaploids (2n = 8x = 72) [4].

Switchgrass has been identified as a promising grass species for forage and biofuel production because of its favorable attributes, such as its perennial growth habits, ability to adapt to a broad range of environments, and its high biomass productivity in marginal land areas. The success of a switchgrass cultivar largely depends on the consistent production of biomass for several years. Studies have been conducted over the past few decades to understand the management practices and the genetic basis that influence biomass production in switchgrass to maximize its utility [5,6]. Sanderson et al. [7] suggested three methods to maximize biomass yield, one of which is to optimize harvest timing and frequency. Conventionally, switchgrass is harvested once per year after the killing-frost to achieve maximum yield for biofuel feedstock production; however, two harvests each year could increase the total biomass yield by exploiting the regrowth potential of the crop [8]. The stability of the biomass yield and regrowth vigor in switchgrass under changing harvest management conditions would help switchgrass growers manage potential fluctuations in the feedstock market and would provide a continuous supply of quality forage for livestock [9]. Switchgrass, similar to many other native warm-season grasses, can produce high-quality forage. When there is prolonged drought for more than two weeks, all other grasses cease to grow, but switchgrass survives through extended periods of drought [10]. Because of its high forage yield and ability to tolerate severe drought compared to other native grasses, switchgrass has earned a reputation as an attractive forage crop, especially in the mid-south of the USA [10]. However, switchgrass is sensitive to frequent defoliation [11,12,13]. Frequent defoliation, or clipping, can cause stand reductions and losses in forage yield. Studies that have been conducted with an aim to optimize harvest frequency and cutting heights in switchgrass suggest that multiple cuttings, especially clipping during fall, would significantly reduce the long-term survival as well as economic return in lowland switchgrass [9,13,14,15]. The long-term survival of any perennial grass depends on its regeneration ability in every growing season. The regeneration ability of perennial grass resides in the dormant crowns and rhizomes (modified underground stems) that produce growing buds at the time of regrowth [16,17]. A number of the inherent properties of switchgrass, including its rhizome dormancy and freezing tolerance, affect the regrowth ability of plants [18,19]. Therefore, rhizome health plays a very significant role in the sustainable production of perennial grasses.

The upland and lowland ecotypes of switchgrass have shown different responses to cutting frequency. A few studies have reported that the cumulative biomass yield per year could be higher under a two-cut system than under a one-cut system, especially in upland cultivars [8,20]. Upland cultivars have more cold hardiness, a shorter growth cycle, and higher photosynthetic rates than lowland cultivars [21]. A greater yield response of upland cultivars with a two-cut system is likely associated with such phenological attributes. Because of the shorter growth cycle, the upland cultivars complete the primary vegetative growth period early in the summer. Therefore, the biomass harvest of the upland cultivars early in the summer allows for a second vegetative growth period during the fall [20]. In contrast, lowland cultivars have a longer vegetative growth period. The lowland cultivars, if clipped in the fall, produce many nonrooted shoots that quickly go dormant as soon as the temperature drops and resume growth the following spring [22]. Nonrooted shoots are vulnerable to damage caused by cold temperatures in the winter, resulting in a reduction in spring growth and overall biomass accumulation in subsequent years. A few other studies suggested that multiple cutting systems may increase the biomass harvest yield in lowland switchgrass, depending on the cultivar and environmental conditions. Mclaughlin et al. [23] reported that a two-cut system improved the biomass yield under favorable weather conditions, whereas the one-cut system was superior in environments where drought was a frequent problem. It has also been documented that multiple cuttings significantly reduce the number of reserves a switchgrass plant could conserve in its below-ground biomass for winter survival and reduces the transfer of adequate energy to the rhizomes for the regrowth in the next growing season [1,17]. Rhizomes are carbohydrate storage organs in many perennial plants and determines regrowth vigor in the next growth cycle. Tao et al. [24] specified that rhizomatousness in rice (*Oryza* spp.) is controlled by several quantitative trait loci (QTL) that have an additive effect. Paterson et al. [25] reported that regrowth after overwintering is associated with rhizomatousness, a polygenic trait, and identified several QTL associated with this trait in johnsongrass (*Sorghum halepense* L.). Sarath et al. [17] reported that QTL associated with rhizomatousness have been identified in monocots such as perennial species of rice (*Oryza sativa*) and sorghum (*Sorghum bicolor*). Additionally, Robins et al. [26] reported that the tetraploid alfalfa (*Medicago sativa* L.) population exhibits continuous variation in regrowth vigor. Based on these studies, it can be hypothesized that the regrowth vigor of switchgrass, especially under flexible harvest management systems, is possibly a quantitative trait and that genotypic variation may also be influenced by the genotype × environment interaction. Phenotypic selection for quantitative traits, especially those having low heritability, is not efficient, as the genotype × environment interaction has a very high confounding effect and often requires laborious phenotyping. To date, little is known about the genetics underlying the response that switchgrass plant may have against multiple cuttings. A few studies that were conducted in the past to evaluate effect of harvest frequency on the biomass yield of switchgrass presented conflicting results that rationalize the need for more research to develop cultivars that are suitable for multiple harvests. The identification of genetic markers and the application of marker-assisted selection would offer a reliable technique to improve quantitative traits such as regrowth vigor, which is critical for a multiple harvest system and for improving biomass yield. In recent times, multi-parent populations, such as the nested association mapping (NAM) population, have emerged as next-generation mapping resources that can be used to understand the genetic basis of quantitatively inherited traits [27]. The NAM population combines the advantages of both association mapping and bi-parental populations and can provide high-power and high-resolution to the QTL detection of complex traits. Therefore, this study was conducted utilizing the NAM population to (i) assess genetic variation and (ii) identify the QTL associated with regrowth vigor after multiple cuttings in lowland switchgrass.

## 2. Results

### 2.1. Analysis of Phenotypic Data

**Figure 1:**
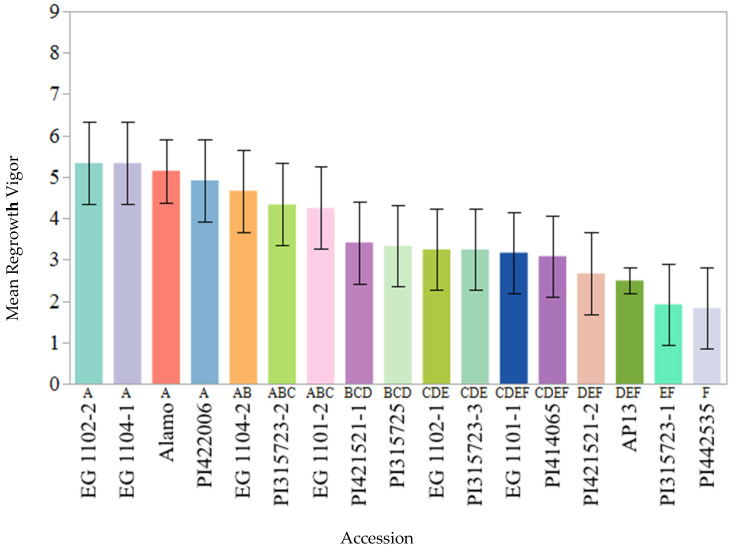
Mean regrowth vigor score (The range of the score is from 0 to 9; 0 = no regrowth, 1 = the least vigorous, 9 = the most vigorous) across two years (2017 and 2018) of the founder parents of the nested association mapping (NAM) population, AP13, and Alamo check. Bars not sharing a common letter are significantly different at *p* < 0.05. Error bars indicate 95% confidence intervals of the mean.

Fifteen founder parents, AP13, and Alamo check exhibited differences in their mean regrowth vigor in the combined data across two years (2017 and 2018) (Figure 1; Table S1), suggesting that these parental accessions possessed a different level of regrowth ability. Three founder parents, EG 1102-2, EG 1104-1, and PI 422006, along with Alamo check exhibited the most superior regrowth vigor compared to others. The founder parent PI 442535 was the least vigorous among all of the parents. The recurrent parent AP13 displayed poor regrowth vigor despite having the same genetic background as Alamo. The mean regrowth vigor of AP13 did not differ from that of founder parent PI 442535.

**Figure 2:**
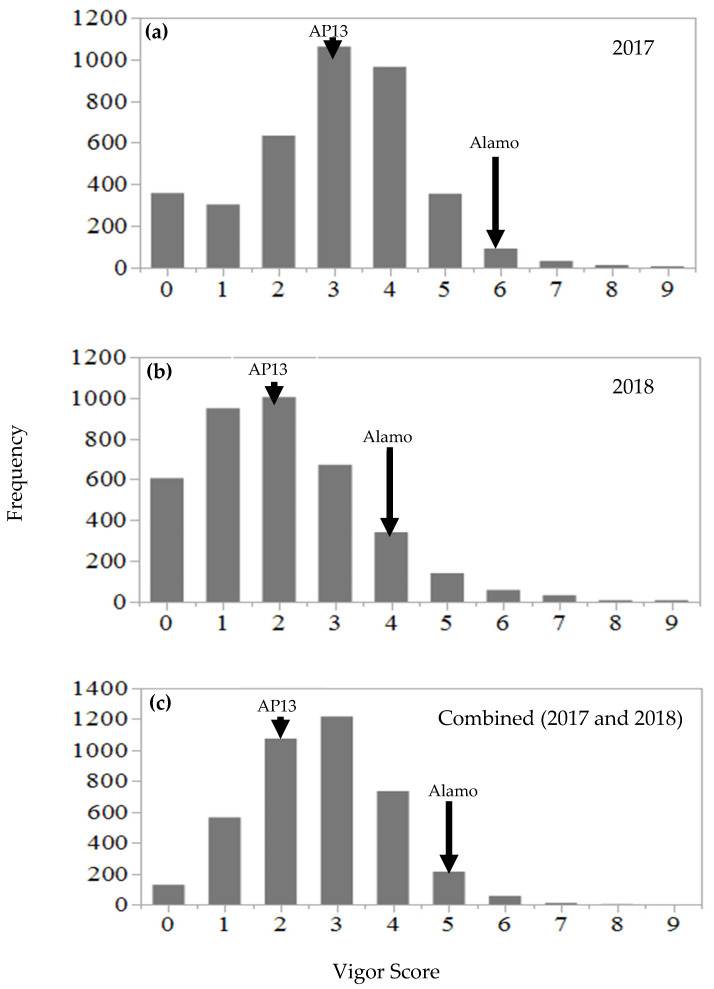
Frequency distribution of regrowth vigor in the nested association mapping (NAM) population for (**a**) 2017, (**b**) 2018, and (**c**) the combined data across two years: 2017 and 2018.

The NAM population displayed nearly continuous variation in regrowth vigor when the data were analyzed across two years (Figure 2). The comparison of 2017 and 2018 data in Figure 2 suggests that the mean regrowth vigor score of the NAM population in 2018 shifted towards the lower score compared to the mean regrowth vigor score in 2017. The analysis of variance of the combined data across two years (2017 and 2018) revealed that the NAM families differed in their mean regrowth vigor (*p* < 0.05) (Table 1). No significant variation in the regrowth vigor was observed among the genotypes within family. It was also evident from the results that the regrowth vigor was influenced by the family × year interaction (*p* < 0.05). The replication effect was not significant, but the genotype within the family × replication interaction was evident. The fixed effect of the year was highly significant (*p* < 0.0001).

The mean for the regrowth vigor and the range across two years are presented in Table 2 and Table S1. The regrowth vigor score of the NAM population ranged from 0 to 9, with a mean score of 2.5. The overall mean regrowth vigor score of the 15 founder parents was 3.6, and it ranged from 0 to 9. The chain-cross parents had a mean regrowth vigor score of 3.5 and a range from 0 to 9. The recurrent parent, AP13, had a mean regrowth score of 2.5, whereas Alamo check had a mean score of 5.1. The mean separation of the combined data revealed that regrowth vigor of the NAM population differed from that of the founder parents, chain-cross parents, and Alamo check (*p* < 0.05). The mean regrowth vigor of the NAM population did not differ from AP13. Likewise, no difference was detected between the mean regrowth vigor of the founder parents and the chain-cross parents. Similar results were observed in 2017. These results were also consistent in 2018, with the exception of that the mean regrowth vigor of the founder parents did not differ from that of Alamo check. The founder and chain-cross parents showed more superior regrowth vigor than AP13 did. The overall mean regrowth vigor score of the NAM population declined by 31% in 2018 compared to in 2017. The decreasing regrowth vigor trend was also observed in founder parents, chain-cross parents, AP13, and Alamo check.

A Spearman rank correlation of the genotypes was calculated for the regrowth vigor and biomass yield and spring emergence of the preceding years (Table 3). The results suggested that regrowth vigor was positively correlated with biomass yield of the preceding years, while it was negatively correlated with the spring emergence in each individual year. The Spearman rank correlation coefficient between the regrowth vigor and biomass yield of previous years ranged from 0.53 to 0.62, whereas the correlation between regrowth vigor and spring emergence ranged from −0.27 to −0.33.

### 2.2. Genetic Linkage Map

A high-density genetic linkage map was constructed using 2684 SNP markers. Details of the genetic linkage maps have been published previously [28]. Briefly, a total of 18 linkage groups were determined. The linkage groups were named according to the name of the chromosomes to which the SNPs were matched with the physical chromosome position on the switchgrass reference genome (*Panicum virgatum* v1.1, DOE-JGI, https://phytozome.jgi.doe.gov/; Accessed on 30 July 2019) [29]. Therefore, the subgenomes were designated as “A” and “B” for each linkage group. It should be noted that newer versions of the maps (*Panicum virgatum* v4.1 and v5.1) have designated the subgenomes as “K” and “N” for each linkage group. A comparison of the older (v1.1) and newer versions (v4.1 and 5.1) of the chromosome names is provided in the supplementary file (Table S2). The map of 18 linkage groups covered a total length of 3119 cM, with individual chromosome sizes ranging from 124 cM (chromosome 8B) to 252 cM (chromosome 4B) (Figure S1 and Table S2). On average, one SNP was mapped every 1.3 cM. The number of markers on the different chromosomes ranged from 69 on chromosome 8A to 273 on chromosome 9B. The map coverage (total genetic distance in cM) is comparable to the coverage of the maps published by Tornqvist et al. [30] and Ali et al. [31] but larger than two other published maps [32,33].

### 2.3. Genomic Regions Associated with Regrowth Vigor

For the individual year datasets, a total of 10 QTL associated with regrowth vigor were identified using composite interval mapping. These QTL were identified on chromosomes 1B, 5A, 6B, and 7B from the 2017 dataset and on chromosomes 5A, 5B, 6B, and 8A from 2018 dataset (Table 4 and Figure S2). The names of each QTL consist of an abbreviation for the trait name (RV = regrowth vigor) followed by the location and year (kn17 = Knoxville, 2017; kn18 = Knoxville, 2018) and the chromosome’s name (Chromosome = 1 to 9, sub-genome = A or B, and a serial number when there were two or more QTL on the same chromosome). From the 2017 data set, one QTL was detected on chromosome 1B, which was called *QRV.kn17.1B* and was found to be linked with marker c1b_53443735, explaining 4.3% of the phenotypic variation for regrowth vigor, with an additive effect of −0.15. On chromosome 5A, three QTL were detected and called *QRV.kn17.5A-1*, *QRV.kn17.5A-2*, and *QRV.kn17.5A-3,* which accounted for a total of 3, 4.7, and 3.1% of the phenotypic variation and were tightly linked with markers c5a_61728698, c5a_9165315, and c5a_8191879, respectively. Two other QTL that were identified from 2017 dataset on chromosomes 6B and 7B were named *QRV.kn17.6B* and *QRV.kn17.7B,* which explained 3.1 and 3.2% of the phenotypic variation, with an additive effect of 0.07 and 0.20, respectively. The nearest markers to *QRV.kn17.6B* and *QRV.kn17.7B* were c6b_20112532 and c7b_17213104, respectively. Similarly, the QTL that were identified from the 2018 dataset on chromosomes 5A, 5B, 6B, and 8A were called *QRV.kn18.5A*, *QRV.kn18.5B*, *QRV.kn18.6B*, and *QRV.kn18.8A,* which explained 3.7, 3.2, 2.6, and 3.8% of the phenotypic variation, respectively. The closest flanking markers of the QTL *QRV.kn18.5A*, *QRV.kn18.5B*, *QRV.kn18.6B*, and *QRV.kn18.8A* were c5a_15120636, c5b_71514101, c6b_3764436, and c8a_14434016, respectively. The additive effect of these four QTL were comparatively similar (0.12 to 0.16).

A total of five QTL were detected using combined data across the years 2017 and 2018. These QTL were called *QRV.kn.1B*, *QRV.kn.5A*, *QRV.kn.6B*, *QRV.kn.7B*, and *QRV.kn.8A*, which explained 1.6 to 3.1% of the phenotypic variation and additive effects ranging from −0.13 to 0.19. All five of these QTL were also detected in either the 2017 or 2018 datasets.

### 2.4. Mode of Gene Action of the Identified QTL

The gene action mode of the identified QTL is presented in Table 4. Out of the 10 QTL that were identified in this study, four displayed a *d/a* ratio in a range from −0.5 to 0.5, indicating additive gene action. Three QTL seemed to exhibit partial dominance (0.5 < *d/a* < 1.25), and three other QTL exhibited over-dominant (*d/a* > 1.25) or under-dominant (*d/a* < −1.25) gene action.

### 2.5. Candidate Gene Search

The homolog-based annotation of candidate genes, which were identified by the scanning sequence flanking 50 kb upstream and downstream of the major QTL peak markers that were obtained from the physical map of the switchgrass genome (*Panicum virgatum* v1.1), is presented in Table 5. Several candidate genes were localized within a 50 kb upstream or downstream region from the peak marker of the identified QTL, with the E-value cutoff 1 × 10^−4^ and sequence similarity above 80%. However, we only presented the one candidate gene that received the best blast hits (the highest identity similarity and the lowest E-value) for each QTL. The QTL on chromosome 1B is associated with Serine/arginine-rich splicing factor RSZ21A (SRSF) and was found to have 90% identity similarity (E-value, 0.00). The candidate gene Phosphoribosylformylglycinamidine synthase was found to have 97% identity similarity with the QTL *QRV.kn17.5A-1* on chromosome 5A. Another QTL on chromosome 5A, *QRV.kn17.5A-2*, was found to have 91% identity similarity (E-value 2 × 10^−58^) with the Bowman–Birk-type wound-induced proteinase inhibitor *W1P1*. The third QTL on chromosome 5A, *QRV.kn17.5A-3,* was found to possess similarity with Probable indole-3-pyruvate monooxygenase YUCCA10, with an identity similarity of 90% (E-value, 0.00). Two other QTL that were identified on chromosome 6B and 7B from the 2017 dataset, *QRV.kn17.6B* and *QRV.kn17.7B*, have 91% identity similarity with the candidate gene AUGMIN subunit 1 (AUG1) and Protein *ECERIFERUM 1* (CER1), respectively. Similarly, four QTL that were found from the 2018 dataset on chromosome 5A, 5B, 6B, and 8A were found to be similar to Probable indole-3-pyruvate monooxygenase YUCCA10 (identity similarity, 90%, and E-value, 0.00), Scarecrow-like protein 9 (identity similarity, 90%, and E-value, 0.00), F-box protein SKIP28 (identity similarity, 95%, and E-value, 5 × 10^−132^), and SUMO-activating enzyme subunit 1A (identity similarity, 83%, and E-value, 9 × 10^−96^), respectively. These candidate genes were scanned using a newer version of the reference genome (*Panicum virgatum* v5.1), and the details are provided in the Supplementary Table S3.

## 3. Discussion

Regrowth vigor, i.e., the ability to regrow soon after the harvesting of the existing stand, is one of the most desirable traits in perennial grasses, such as switchgrass, especially for long term production under changing harvest management systems. In this study, significant genetic variation was observed for regrowth vigor. The founder parents EG 1102-2, EG 1104-1, and PI 422006 showed the most vigorous regrowth. These cultivars originate from the south to southeastern region of the United States, and possibly favorable genes were constitutively expressed in the southeastern climate of Knoxville. The founder parent PI 442535 was the least vigorous among all of the parents, which could be due to the fact that it is an exotic cultivar introduced from Belgium and not well-adapted to the study region. Alamo was the genotype that showed the most vigorous regrowth across different years the most consistently. Alamo switchgrass was originally selected for use in pastures [34], which may have resulted in indirect selection for regrowth vigor. Interestingly, recurrent parent AP13 (PI 671956; https://npgsweb.ars-grin.gov/; Accessed on 21 October 2021) [35] displayed poor regrowth vigor despite possessing the same genetic background as Alamo. AP13 was originally selected for high phosphorus (*p*) uptake from the lowland cultivar, Alamo. The literature suggests that the genotypes with a lower P uptake rate might be compensated for by larger root systems to explore more soil for P foraging [36,37]. Perhaps such morphological changes in the root system could have led to more carbohydrate reserves in the root system of lower P uptake genotypes in contrast to higher P uptake genotypes. Morphological changes in the root system of the genotypes with higher P uptake were reported in sorghum (*Sorghum bicolor* L.) [36] and maize (*Zea mays* L.) [37]. In switchgrass, it is known that the reserves that accumulate in the roots directly affect rhizome health [17]. Selection for higher P uptake may have affected the root architecture in AP13 and depleted regrowth vigor.

The NAM population displayed nearly continuous variation in the regrowth vigor when the data were analyzed across two years (Figure 2), which suggests the polygenic effect that is associated with regrowth vigor. The overall mean regrowth vigor score of the NAM population declined by 31% in 2018 compared to in 2017. The decreasing regrowth vigor trend was also observed for the founder parents, chain-cross parents, AP13, and Alamo check. Such a decline in the regrowth vigor is likely associated with the late-season clipping in 2018 and the poor reserve accumulation in the root from the previous year. It should be noted that in this experiment, plant biomass was harvested under a one-cut system until 2016, and thereafter, a two-cut system was implemented in 2017 and 2018 to evaluate the regrowth vigor. Because of the two-cut system, the plants could not complete a full growth cycle in 2017. As discussed earlier, the plant stand from the previous year is important in order for any perennial grass to accumulate reserves in the root, which is required for regrowth during the next growing season.

The analysis of variance of the combined data across the two years (2017 and 2018) revealed that the NAM families differed in terms of their mean regrowth vigor (*p* < 0.05), suggesting that the regrowth ability of a few genotypes declined over the year, especially under the system with two clippings per year. A study pointed out that switchgrass can tolerate a single clipping almost anytime and with no year-to-year reduction in plant vigor; however, two or more clippings per year could reduce crown survival and plant vigor [13]. The year-to-year decline in regrowth vigor under a multiple harvest system, as observed in this study, is not surprising because selection for superior regrowth ability under a multiple-harvest system has not been attempted in the past. The significant variation in the regrowth vigor among the NAM families also indicates the role of additive genes for controlling regrowth vigor. As mentioned earlier, no significant variation in the regrowth vigor was observed among the genotypes within each family. However, regrowth vigor was influenced by family × year interaction (*p* < 0.05), which means that the families that had superior regrowth vigor in the initial year would not necessarily hold the highest ranking in subsequent years. Such flip-flopping is not surprising due to the fact that variations in the temperature and precipitation between years could affect switchgrass growth. The effect of temperature and precipitation on switchgrass growth has been well documented [38,39,40]. No difference was observed between replication, but within-family genotype × replication interaction was evident. Such within-family genotype × replication interaction could be due to the fact that the genotypes in replication I and II received different clipping frequencies. As mentioned earlier, replication I was planted a year earlier and thus received more clippings than replication II. Regrowth vigor data were recorded after five and seven clippings during replication I compared to four and six clippings in replication II. We also observed that the year had a significant affect (*p* < 0.01), which could be associated with the reduction in the regrowth vigor and the subsequent mortality of some of the plants during the next growing season under the two-cutting system. Several studies have indicated that switchgrass is sensitive to multiple cuttings [11,12,13] given the fact that the number of reserves accumulated in the roots would directly affect rhizome health and switchgrass regrowth during the next growing season [17].

A comparison of the regrowth vigor means revealed that both the founder parents and chain-cross parents showed more superior regrowth vigor than AP13. This suggests an heterosis event due to complete dominance, as the chain-cross parents originated from a cross of the AP13 × the founder parents and showed regrowth vigor that was similar to that of the founder parents but still superior to AP13. However, heterosis was abruptly exhausted in the NAM population, resulting in poor regrowth vigor compared to both the founder parent and the chain-cross parent. One explanation for this is that it may be the case due to sib mating. Further, the C-scaling test (4F_2_-2F_1_-P_1_-P_2_ = 0) revealed that the additive–dominance model of inheritance is not adequate to explain these data, which indicates that the genetic complexity that is associated with regrowth vigor is possibly due to non-allelic interaction or abnormal chromosome behavior. In the future, an in-depth study in the heterosis effect and the role of non-additive genes would be beneficial for improving regrowth vigor.

The correlation analysis suggests that plants with a higher biomass yield and earlier emergence in the spring have better regrowth vigor in succeeding years. This indicates that the combination of two traits, i.e., genotypes with a high turnover of biomass yield during the initial year of growth and earlier spring emergence can be used for the indirect selection of high regrowth vigor. Razar and Missaoui [18] reported the direction of the linear relationship between fall regrowth height, biomass yield, and spring emergence, and their results were similar to our observations; however, their results showed a slight difference in the strength of the relationships.

Among all of the QTL identified in this study, only two of the QTL on chromosomes 5A and 6B were stably detected in individual year data as well as in the combined data across the years, with a slight shift in position. The QTL on chromosomes 1B, 7B, and 8A were detected in either 2017 or 2018; however, it was observed that the marker peak just failed to exceed the LOD threshold level in the alternative year. This indicates that the QTL × year interaction has a strong effect on regrowth vigor. Indeed, our results showed that the family means were influenced by family × year interaction (Table 1). No QTL was identified to possess a large effect. This suggests that the regrowth vigor is a complex trait and that it might be influenced by genotype × environment interaction. Environmentally responsive QTL were reported in several studies in the past, such as for growth related traits, biomass yield and plant height in switchgrass (*Panicum virgatum* L.) [31,41], and regrowth in sorghum (*Sorghum bicolor* L.) [42]. Other studies in different species, such as in rice (*Oryza sativa* and *O. longistaminata*) [24], sorghum (*Sorghum bicolor* and *S. propinquum*) [43], alfalfa (*Medicago sativa* L) [26], and wild relatives of maize (*Zea mays* ssp. *parviglumis* and *Z. diploperennis*) [44], have reported the existence of QTL via environment interactions, multiple minor effects, QTLwith small effects distributed over several genomic regions, and both male and female parents contributing favorable alleles associated with regrowth ability. These studies reflect the level of complexity associated with this trait. Looking at the mode of gene action of the identified QTL, we learned that 6 out of the 10 QTL that were identified in this study exhibited either partial dominance or overdominance. The dominance effect of these QTL indicates that regrowth vigor is possibly influenced by the interactions between individual alleles, and this relates to the advantage of heterozygosity. This result suggests that heterosis breeding has a great scope for improving regrowth vigor in switchgrass. The potential of heterosis breeding in switchgrass for biomass yield was demonstrated in the past [45,46].

We identified ten candidate genes that were localized within 50 kb upstream or downstream from the peak markers of the identified QTLs. The candidate genes that were found in this study hold similarities to the closely related species foxtail millet (*Setaria italica*), Sorghum (*Sorghum bicolor*), and Hall’s Panicgrass (*Panicum hallii*), which signifies the reliability of a similar gene function in switchgrass [32]. Among these genes, the Serine/arginine-rich splicing factor RSZ21A (SRSF) is located on chromosome 1B and is associated with the QTL *QRV.kn17.1B*. Duque [47] described that SRSF plays a key role in the regulation of gene expression that is important for adaptation to physiological and environmental stress. The candidate gene Phosphoribosylformylglycinamidine synthase was found to be associated with *QRV.kn17.5A-1*, which is essential for de novo purine nucleotide biosynthesis [48]. Another gene, Bowman–Birk-type wound-induced proteinase inhibitor *W1P1,* which is related to a plant defense mechanism against pathogens or physical injury [49], was found to be associated with the second QTL found on chromosome 5A, *QRV.kn17.5A-2*. The gene Probable indole-3-pyruvate monooxygenase YUCCA10 was found on the third QTL of chromosome 5A, *QRV.kn17.5A-3*. Two other genes, AUGMIN subunit 1 (AUG1) and Protein *ECERIFERUM 1* (CER1), were found to be associated with *QRV.kn17.6B* and *QRV.kn17.7B*, respectively. It has been understood that AUG1 plays a critical role in microtubule organization during cell division, whereas CER1 is linked to responses to biotic and abiotic stresses [50,51]. Similarly, four genes, Probable indole-3-pyruvate monooxygenase YUCCA10, Scarecrow-like protein 9, F-box protein SKIP28, and SUMO-activating enzyme subunit 1A, were found to be associated with the QTL found in the 2018 dataset on chromosomes 5A, 5B, 6B, and 8A, respectively. Among these genes, Probable indole-3-pyruvate monooxygenase YUCCA10 has been reported to be involved in auxin synthesis, which affects leaves and flower formation [52]. Scarecrow-like protein 9 is known to play an important role in development and to cope with either biotic or abiotic stress in plants [53]. F-box protein SKIP28 is considered to play a variety of roles in developmental processes, including plant hormonal signal transduction, secondary metabolism, senescence, and responses to both biotic and abiotic stresses [54]. SUMO-activating enzyme subunit 1A mediates the activation of SUMO (small ubiquitin-related modifier) proteins, which coordinate the gene expression that is necessary for the development and hormonal and environmental responses of plants [55]. In the future, elucidating the functional effect of these candidate genes by gene expression analysis and manipulating these genes may help to develop switchgrass cultivars for improved regrowth vigor.

## 4. Materials and Methods

### 4.1. Plant Materials

A Nested Association Mapping (NAM) population of switchgrass, which had been developed at the Nobel Research Institute (Ardmore, OK, USA), was used in this study. The NAM population was developed by crossing 15 diverse lowland switchgrass genotypes to a common parent, “AP13”. Details of the genetic backgrounds of these switchgrass genotypes and a schematic diagram of the NAM population development process can be accessed in our previously published article [28]. In brief, 15 diverse genotypes were used as the pollen parents, and AP13 was used as a recurrent parent. AP13 was used as a recurrent parent because its genome has been sequenced and is widely used in switchgrass genomics research. Additionally, it ensured a consistent maternal effect across all of the crosses. To make the crosses, AP13 was clonally propagated, and a copy was grown alongside each of the paternal genotypes in a greenhouse under a regime of a 32/21 °C day/night temperature and a 16 h photoperiod. Upon flowering, the inflorescences from each pair of parents were bagged together, and the seeds of the resulting crosses were harvested separately. From each resulting F_1_ family, 10 F_1_ plants were raised and chain-crossed with one another to generate recombinant chain-cross families. During the chain cross process, the inflorescence of the first and the second F_1_ plants were bagged together, and then second and third F_1_ plants were bagged together, and so on, with the final bagging taking place between the tenth and the first F_1_ plants. Thus, 10 recombinant chain-cross families within each of the 15 F_1_ families were generated. Twenty random plants were selected from each chain cross to generate 200 chain-cross progenies (10 chain-cross × 20 plants = 200). However, eight “AP13 × diverse genotype” F_1_ families out of fifteen did not produce the required number of F_1_ seedlings; thus, only 5 F_1_ plants were chain-crossed, and 15 random plants were selected from each chain-cross to generate 75 chain-cross progenies (5 chain-cross × 15 plants = 75). The chain-cross progenies are hereafter referred to as the “pseudo F_2_ progenies” that created the “NAM Population”. Therefore, the NAM population comprised 2000 randomly selected pseudo F_2_ progenies (Table 6). The 15 diverse genotypes that were used as pollen parents to make crosses with AP13 are hereafter referred to as “founder parents”. Similarly, the F_1_ plants that were generated from each of the AP13 × diverse genotype crosses and used in the chain cross to generate pseudo F_2_ progenies are hereafter referred to as “‘chain-cross parents”. A total of 2350 plants, including 2000 pseudo F_2_ progenies (NAM population), 2 copies of each chain-cross parents (2 × 135 = 270), 3 copies of each founder parent (3 × 15 = 45), 30 copies of AP13, and 5 copies of Alamo check, were evaluated in this study.

### 4.2. Field Experiment and Phenotypic Data Collection

The NAM population experiment was originally established for a US Department of Energy (DOE)-funded project (Grant #DE-SC0008781) to assess biomass yield and other feedstock traits. The NAM population along with the chain-cross parents, founder parents, AP13, and Alamo check were planted at the Plant Science Unit (35°54′1″ N 83°57′17″ W) of the East Tennessee Research and Education Center (ETREC), Knoxville, TN, USA, which is located at an elevation of 259 m. The soil type is a Shady loam (fine-loamy, mixed, subactive, thermic Typic Hapludults). The field experiment was planted in two replications using an alpha lattice design, with plant-to-plant spacing at 0.9 m. In each replication, the plant materials were accommodated in a block of 47 rows with 50 plants in each row. To minimize border effects, border plants were planted all around the main plots. Because of the insufficient number of ramets produced in 2013, only one replication was planted in 2013 (June), while the other replication was established in 2014 (July). The field nursery was treated with the pre-emergence herbicides Dual II Magnum (Metolachlor; Syngenta Crop Protection, Inc., Greensboro, NC, USA) at the rate of 2.84 L ha^−1^ and Prowl H_2_O (Perdamethalin; BASF Corporation, Research Triangle Park, NC, USA) at the rate of 3.31 L ha^−1^ during the spring of each year until 2017. A post-emergence herbicide, 2,4-D was applied approximately 60 days post transplanting of field nursery, at the rate of 2.37 L ha^−1^ with a surfactant at the rate of 1.18 L ha^−1^. The field nursery was not supplemented with any fertilizer during the establishment year. During the post-establishment years, the field nursery was amended with 60 kg ha^−1^ N each spring until 2017.

**Figure 3:**
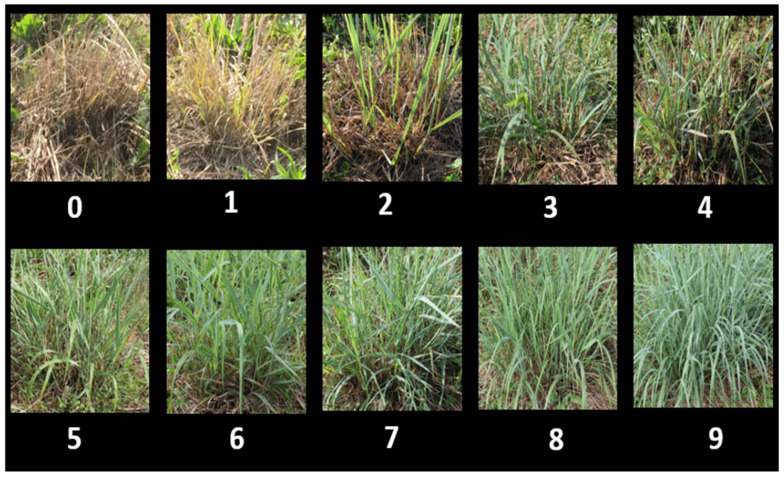
Regrowth vigor scoring scale (0 to 9 rating scale: 0 = no regrowth, 1 = the least vigorous, 9 = the most vigorous).

The plant materials were harvested under a one cut system from 2013 to 2016 to fulfil research objectives for the DOE-funded project. Afterwards, the plant materials were clipped twice per year to understand the genetics underlying regrowth vigor under multiple harvest management systems. In the year of 2017, the plants were clipped in July, and regrowth vigor was recorded in August 2017, i.e., 30 days after clipping. After recording the 2017 regrowth vigor data, the plants were clipped again in November. In 2018, the plants were clipped in August, and regrowth vigor was recorded in September 2018. The regrowth vigor of each plant was recorded using a scale from 0 to 9 (0 = no regrowth, 1 = the least vigorous regrowth, 9 = the most vigorous regrowth) (Figure 3) from each replication in each year. The data from replication 1 represent the regrowth vigor of the population after the fourth and fifth year of planting (or after five and seven clippings), while data from replication 2 represent regrowth vigor after the third and fourth year of planting (or after four and six clippings).

Spring emergence and biomass yield data, which were recorded in 2015 and 2016, were used for the correlation analysis. The spring emergence data were recorded as the number of Julian calendar days, beginning on the first day (January 1) of each year. For the biomass yield data, the plants were harvested under a one-cut system after the killing-frost, and the harvest weight was recorded in kg.

### 4.3. Genotyping and Linkage Map Construction

Genomic DNA was extracted from lyophilized young leaves using the MagAttract 96 DNA Plant Core Kit (Cat# 67165; Qiagen Inc., Valencia, CA, USA). The NAM population was genotyped using the same exome-capture method as the one described in Evans et al. (2014) [56]. A two-step process was involved in the genotyping and linkage map construction of the NAM population. In the first step, a total of 540,783 SNPs specific to the NAM population were identified from the genomic sequence data generated using the exome-capture method. The resulting sequences were aligned with the switchgrass reference genome, AP13 (*Panicum virgatum* v1.1, DOE-JGI, https://phytozome.jgi.doe.gov/; Accessed on 30 July 2019) [29]. Furthermore, the SNPs were filtered based on missing data points and segregation patterns. SNPs were not included for linkage analysis if the missing data points exceeded a threshold of 30% and if the SNPs were significantly distorted from the 1:2:1 segregation ratio based on the χ^2^ goodness-of-fit tests for the F_2_ population. In the second step, a total 13,451 SNPs were used to construct linkage maps meeting the filtering criteria and that were also present across all of the founder parents covering 18 chromosomes. Linkage map construction was performed in JoinMap v4.1 (Kyazma, Wageningen, Netherlands) by employing the maximum likelihood method and Kosambi mapping function [57] to convert the recombination distance between the markers into centimorgan (cM) map units. Eighteen linkage groups were identified for each subpopulation, and a final map was created by joining the same linkage group from each subpopulation. The final linkage map is provided in Figure S1 and comprises 2684 SNPs distributed across 18 linkage groups.

### 4.4. Phenotypic Data Analysis

Phenotypic data analysis was performed using MIXED model analysis (PROC MIXED) in SAS 9.4 (SAS Institute, Cary, NC, USA). In the data analysis model, the year was considered fixed, whereas the replication, family, and genotypes within the family were considered random. Least square means of individual genotypes across replications were obtained in a separate model considering the genotype as a fixed effect, and their statistical differences were detected using Fisher’s protected LSD (*p* < 0.05). Spearman’s rank correlation analysis was conducted in JMP Pro 15 (SAS Institute, Cary, NC, USA) to examine the linear relationships between regrowth vigor and spring emergence and biomass yield from the previous year.

### 4.5. QTL Analysis and Candidate Gene Search

A QTL analysis of the regrowth vigor was performed separately on the data from 2017 and 2018. For the QTL analysis, the mean values of two replications were calculated for each year. The QTL analysis was performed using the composite interval mapping (CIM) method in WinQTL Cartographer Ver. 2.5 [58]. The CIM was run using standard model 6 with five markers as a control in a forward regression model. The window size and walking speed were 10 cM and 1 cM, respectively. The genome-wide threshold for the logarithm of odds (LOD) values (*p* < 0.05) were determined via 1000 permutation tests on the datasets for each year as implemented by the WinQTL Cartographer program. In this study, a LOD score threshold ≥ 3, which could be derived from the permutation test, was used to declare a putative QTL. The relative magnitude of the effect of each significant QTL was estimated as the percentage of the phenotypic variation explained (PVE) by the QTL. For the candidate gene search, we scanned 50 kb up- and down-stream regions of the tightly linked SNPs, as carried out by Ali et al. (2019) [31]. Briefly, the transcript sequence flanking the 50 kb up- and down-stream regions of the SNPs tightly linked to the QTL peak was retrieved from the physical map of the switchgrass reference genome (*Panicum virgatum* v1.1; DOE-JGI; https://phytozome.jgi.doe.gov/; Accessed on 30 July 2019) [29] and was subjected to a Blast search in the NCBI database under the expected value threshold of <10^−4^ and identity similarity >80%.

## 5. Conclusions

In summary, a notable variation in the regrowth vigor among the NAM families was observed, demonstrating the opportunity to improve this trait by exploiting additive genes. Our study suggests that the regrowth vigor of switchgrass is a complex trait that involves both additive and dominant gene action. The QTL analysis revealed ten significant genomic regions that were associated with regrowth vigor. The majority of the QTL that were identified in this study showed dominant and over-dominant gene action, indicating that these QTL have a heterozygous advantage. However, the population used in this study (pseudo F_2_) had less power for estimating additive effects but more power for estimating dominant effects. This study takes a step forward in understanding the genetics underlying regrowth vigor in switchgrass after multiple harvests. In the future, studies focused on validating the functional effects of the markers and candidate genes identified in this study could provide great leads in cultivar improvements in terms of high regrowth vigor in lowland switchgrass through molecular breeding.

## Figures and Tables

**Table 1:** Variance components and tests of fixed effects due to year of the nested association mapping (NAM) population for regrowth vigor.

Sources of Variation	2017	2018	Combined
	Variance Component		
Rep ^†^	0.09	0.05	0.05
Family	0.12 **	0.09 **	0.08 *
Genotype (Family)	-	0.53 ***	-
Family × Year	-	-	0.02 *
Genotype (Family) × Rep	-	-	1.82 ***
Residual	2.52 ***	2.12 ***	0.62 ***
	**Test of fixed effects (F-values)**		
Year	-	-	302.9 ***

* Significant at *p* < 0.05, ** significant at *p* < 0.01, *** significant at *p* < 0.0001; ^†^ Rep, replication.

**Table 2:** Summary statistics for the regrowth vigor ^†^ of the nested association mapping (NAM) population, founder parents, chain-cross parents, AP13, and Alamo check.

Population	2017			2018			Combined		
	Mean	Min	Max	Mean	Min	Max	Mean	Min	Max
NAM	2.9 (1.6) ^a^	0	9	2.0 (1.6) ^a^	0	9	2.5 (1.6) ^a^	0	9
Founder Parents	4.0 (2.2) ^b^	0	9	3.3 (2.4) ^bc^	0	9	3.6 (2.3) ^b^	0	9
Chain-cross Parents	4.0 (1.9) ^b^	0	9	3.0 (1.9) ^b^	0	9	3.5 (2.0) ^b^	0	9
AP13	2.8 (1.5) ^a^	-	-	2.1 (1.4) ^a^	-	-	2.5 (1.5) ^a^	-	-
Alamo	6.1 (2.2) ^c^	-	-	4.2 (2.5) ^c^	-	-	5.1 (2.5) ^c^	-	-

^†^ Regrowth vigor (the range of the score is from 0 to 9: 0 = no regrowth, 1 = the least vigorous, 9 = the most vigorous plant). Within the column of the mean, a value in parenthesis displays standard deviation, and different letter groupings denote a significant difference in the mean (*p* < 0.05).

**Table 3:** Spearman’s rank correlation (*ρ*) between regrowth vigor (The range of the score is from 0 to 9: 0 = no regrowth, 1 = the least vigorous, 9 = the most vigorous), spring emergence (Julian calendar days), and biomass yield (kg).

Trait	Regrowth Vigor 2017	Regrowth Vigor 2018	Spring Emergence 2015	Spring Emergence 2016	Biomass Yield 2015
Regrowth Vigor 2018	0.73 ***				
Spring Emergence 2015	−0.28 ***	−0.27 ***			
Spring Emergence 2016	−0.29 ***	−0.33 ***	0.30 ***		
Biomass Yield 2015	0.56 ***	0.53 ***	−0.30 ***	−0.37 ***	
Biomass Yield 2016	0.61 ***	0.62 ***	−0.23 ***	−0.39 ***	0.81 ***

*** Significant at *p* < 0.0001.

**Table 4:** Quantitative trait loci (QTL) position, single nucleotide polymorphism (SNP) marker, logarithm of the odds (LOD), additive effect (AE), ratio of the dominance to additive effect, and phenotypic variation explained (PVE) for QTL associated with regrowth vigor identified by composite interval mapping (CIM) in the nested association mapping (NAM) population.

QTL	Chr ^‡^	Environment	Position	Nearest SNP	LOD	AE ^¶^	*d/a* ^†^	PVE (%)
*QRV.kn17.1B*	1B	2017	120	c1b_53443735	3.6	−0.15	−0.46	4.3
*QRV.kn17.5A-1*	5A	2017	34.2	c5a_61728698	4.0	0.12	2.08	3.0
*QRV.kn17.5A-2*	5A	2017	140.6	c5a_9165315	4.1	−0.15	−1.4	4.7
*QRV.kn17.5A-3*	5A	2017	169.1	c5a_15120636	8.3	0.26	0.38	3.1
*QRV.kn17.6B*	6B	2017	72.1	c6b_20112532	3.8	0.07	3.57	3.1
*QRV.kn17.7B*	7B	2017	47.8	c7b_17213104	4.9	0.20	1.00	3.2
*QRV.kn18.5A*	5A	2018	168.1	c5a_15120636	3.3	0.16	0.37	3.7
*QRV.kn18.5B*	5B	2018	165	c5b_71514101	3.2	0.12	1.08	3.2
*QRV.kn18.6B*	6B	2018	48	c6b_3764436	4.5	0.16	1.00	2.6
*QRV.kn18.8A*	8A	2018	86.7	c8a_14434016	3.1	0.14	0.01	3.8
*QRV.kn.1B*	1B	Combined	120	c1b_53443735	3.4	−0.13	−0.61	3.1
*QRV.kn.5A*	5A	Combined	168.1	c5a_15120636	5.5	0.19	0.52	3.0
*QRV.kn.6B*	6B	Combined	48	c6b_3764436	4.1	0.15	0.86	1.6
*QRV.kn.7B*	7B	Combined	47.8	c7b_17213104	4.3	0.15	0.98	2.3
*QRV.kn.8A*	8A	Combined	86.7	c8a_14434016	3.4	0.16	0.14	2.9

^‡^ Chr, chromosome. ^¶^ negative AE values indicate that the favorable allele is derived from the recurrent parent AP13. ^†^ *d/a*, ratio of the dominance to additive effect, d = dominance effect, a = additive effect.

**Table 5:** Homolog-based annotation of candidate genes localized within the range of 50 kb upstream and downstream to the linked marker of the identified QTL associated with regrowth vigor in switchgrass.

QTL.	Nearest SNP	Homolog-Based Annotation of Candidate Gene	Homolog Description (Reference Sequence, Species)	Identity Similarity	E-Value
*QRV.kn17.1B*	c1b_53443735	Serine/arginine-rich splicing factor RSZ21A	XM_004954123.4, *Setaria italica*	90%	0.00
*QRV.kn17.5A-1*	c5a_61728698	Phosphoribosylformylglycinamidine synthase	XM_025959288.1, *Panicum hallii*	97%	0.00
*QRV.kn17.5A-2*	c5a_9165315	Bowman–Birk type wound-induced proteinase inhibitor *W1P1*	XM_002457398.2, *Sorghum bicolor*	91%	2 × 10^−58^
*QRV.kn17.5A-3*	c5a_15120636	Probable indole-3-pyruvate monooxygenase YUCCA10	XM_004967692.3, *Setaria italica*	90%	0.00
*QRV.kn17.6B*	c6b_20112532	AUGMIN subunit 1	XM_004972433.2, *Setaria italica*	91%	0.00
*QRV.kn17.7B*	c7b_17213104	Protein *ECERIFERUM 1*	XM_002448115.2, *Sorghum bicolor*	91%	1 × 10^−141^
*QRV.kn18.5A*	c5a_15120636	Probable indole-3-pyruvate monooxygenase YUCCA10	XM_004967692.3, *Setaria italica*	90%	0.00
*QRV.kn18.5B*	c5b_71514101	Scarecrow-like protein 9	XM_004970484.3, *Setaria italica*	90%	0.00
*QRV.kn18.6B*	c6b_3764436	F-box protein SKIP28	XM_004973424.2, *Setaria italica*	95%	5 × 10^−132^
*QRV.kn18.8A*	c8a_14434016	SUMO-activating enzyme subunit 1A	XM_002449480.2, *Sorghum bicolor*	83%	9 × 10^−96^

**Table 6:** Founder parents included in the development of the nested association mapping (NAM) population and number of subsequent pseudo F_2_ progenies.

Founder Parent	Chain-Cross Parent (Pseudo F_1_s)	Pseudo F_2_ Progenies
PI414065	F_1_-1	75
PI442535	F_1_-2	200
PI421521-1	F_1_-3	75
PI421521-2	F_1_-4	200
PI315725	F_1_-5	75
PI315723-1	F_1_-6	200
PI315723-2	F_1_-7	200
PI315723-3	F_1_-8	200
PI422006	F_1_-9	75
EG 1101-1	F_1_-10	75
EG 1101-2	F_1_-11	75
EG 1102-1	F_1_-12	75
EG 1102-2	F_1_-13	200
EG 1104-1	F_1_-14	200
EG 1104-2	F_1_-15	75
		Total: 2000

Note: Each founder parent was crossed with a common recurrent parent “AP13” to generate pseudo F_1_s.

## Data Availability

The data are contained within the article and supplementary materials. Additional data presented in this study are available on request from the corresponding author.

## Supplementary Materials

The following supporting information can be downloaded at: https://www.mdpi.com/article/10.3390/plants11040566/s1, Figure S1: Linkage map comprised of 2684 single nucleotide polymorphism (SNP) markers; Figure S2: Quantitative trait loci (QTL) associated with the regrowth vigor identified by composite interval mapping (CIM) using the nested association mapping (NAM) population; Table S1: Summary statistics for the regrowth vigor (The range of the score is from 0–9; 0 = no regrowth, 1 = the least vigorous, 9 = the most vigorous) of the NAM families, Alamo check, AP13, and founder parents; Table S2: Single nucleotide polymorphism (SNP) markers and their positions on chromosome; Table S3: Localization of the identified candidate genes using *Panicum virgatum* v5.1 reference map.
