# Supplementary material for: Identification of QTL Associated with Regrowth Vigor Using the Nested Association Mapping Population in Switchgrass
Source: Plants (Basel). 2022 Feb 21;11(4):566. doi: 10.3390/plants11040566 (PMC8874488; doi:10.3390/plants11040566)
Supplement: Supplementary file 1 [file plants-11-00566-s001.zip › Figure S2_QTL.pdf]

Figure S2. Quantitative trait loci (QTL) associated with regrowth vigor identified by Composite Interval Mapping (CIM) using the Nested Association Mapping (NAM) population.

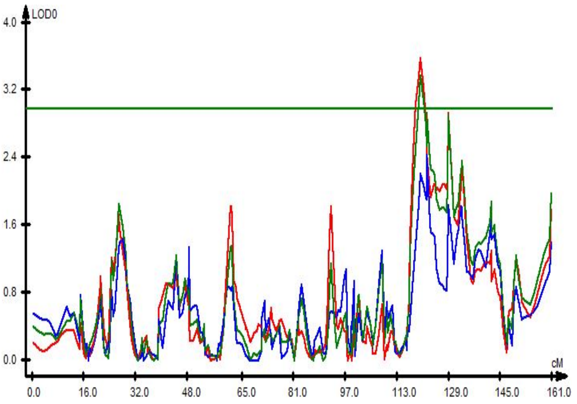

Chromosome 1B

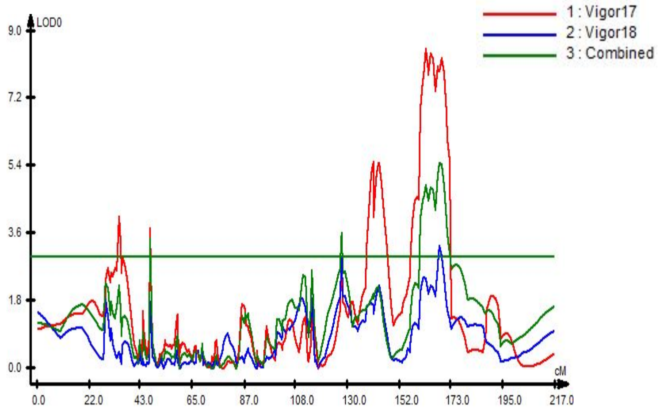

Chromosome 5A

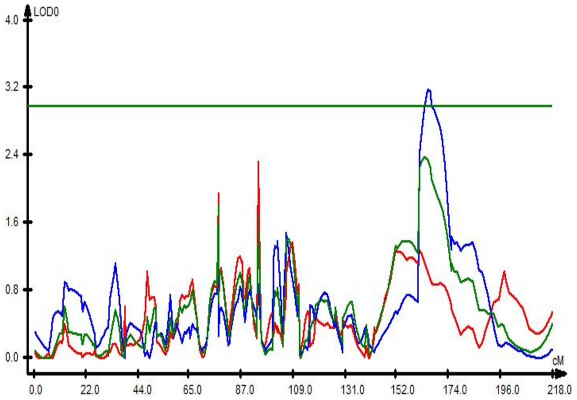

Chromosome 5B

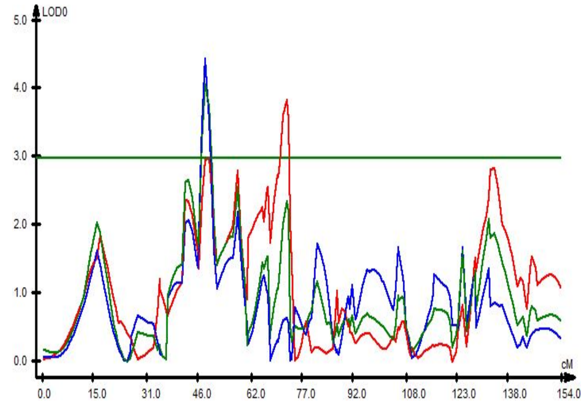

Chromosome 6B

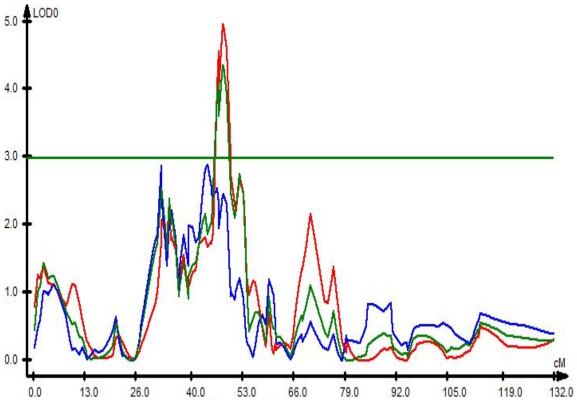

Chromosome 7B

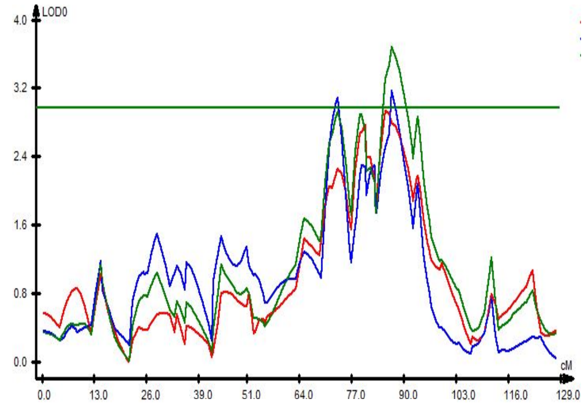

Chromosome 8A
