# Supplementary figures and images for: Identification of QTL Associated with Regrowth Vigor Using the Nested Association Mapping Population in Switchgrass
Source: Plants (Basel). 2022 Feb 21;11(4):566. doi: 10.3390/plants11040566 (PMC8874488; doi:10.3390/plants11040566)

Figure S1. Linkage map comprising 2684 single nucleotide polymorphism (SNP) markers.

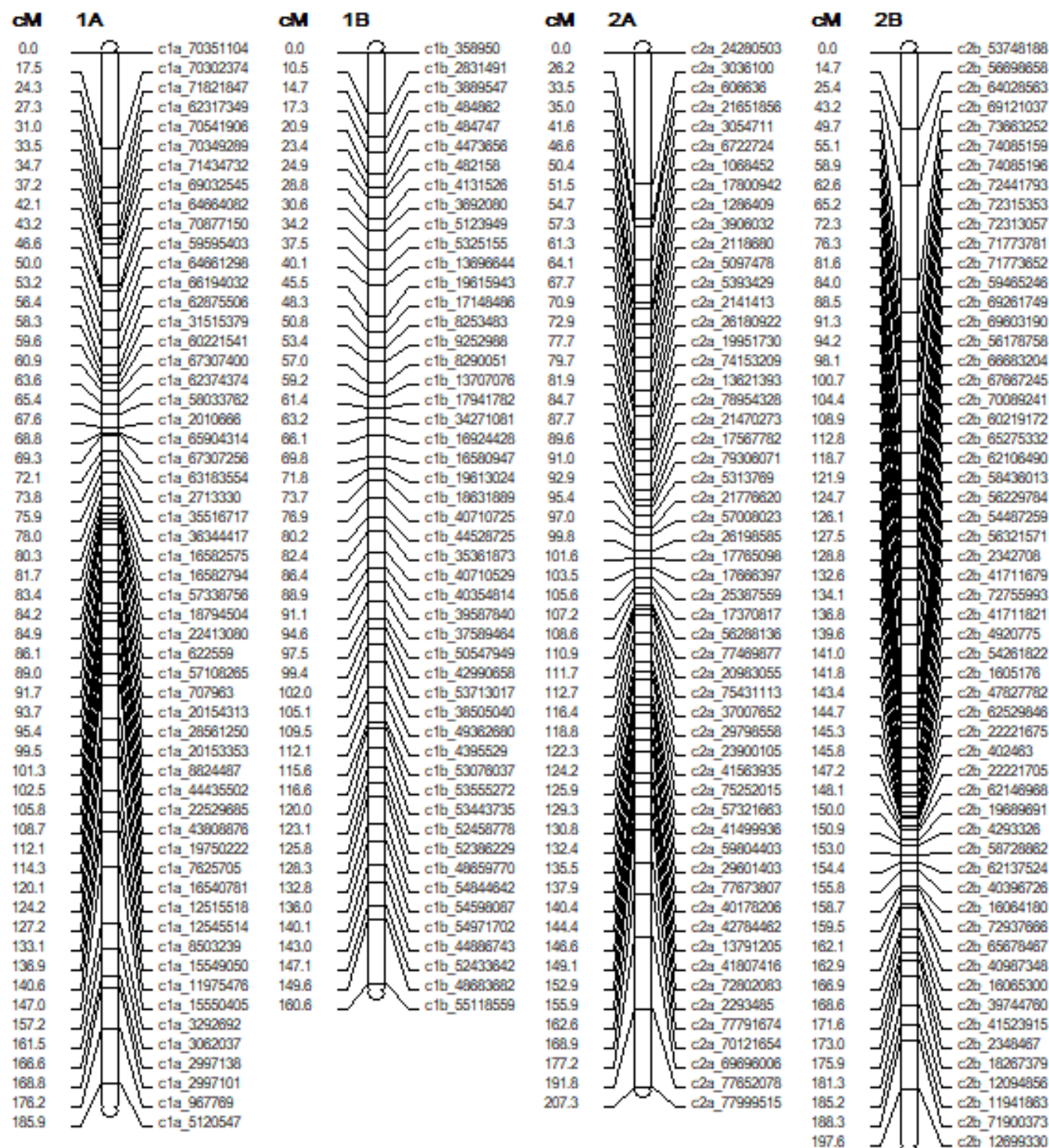

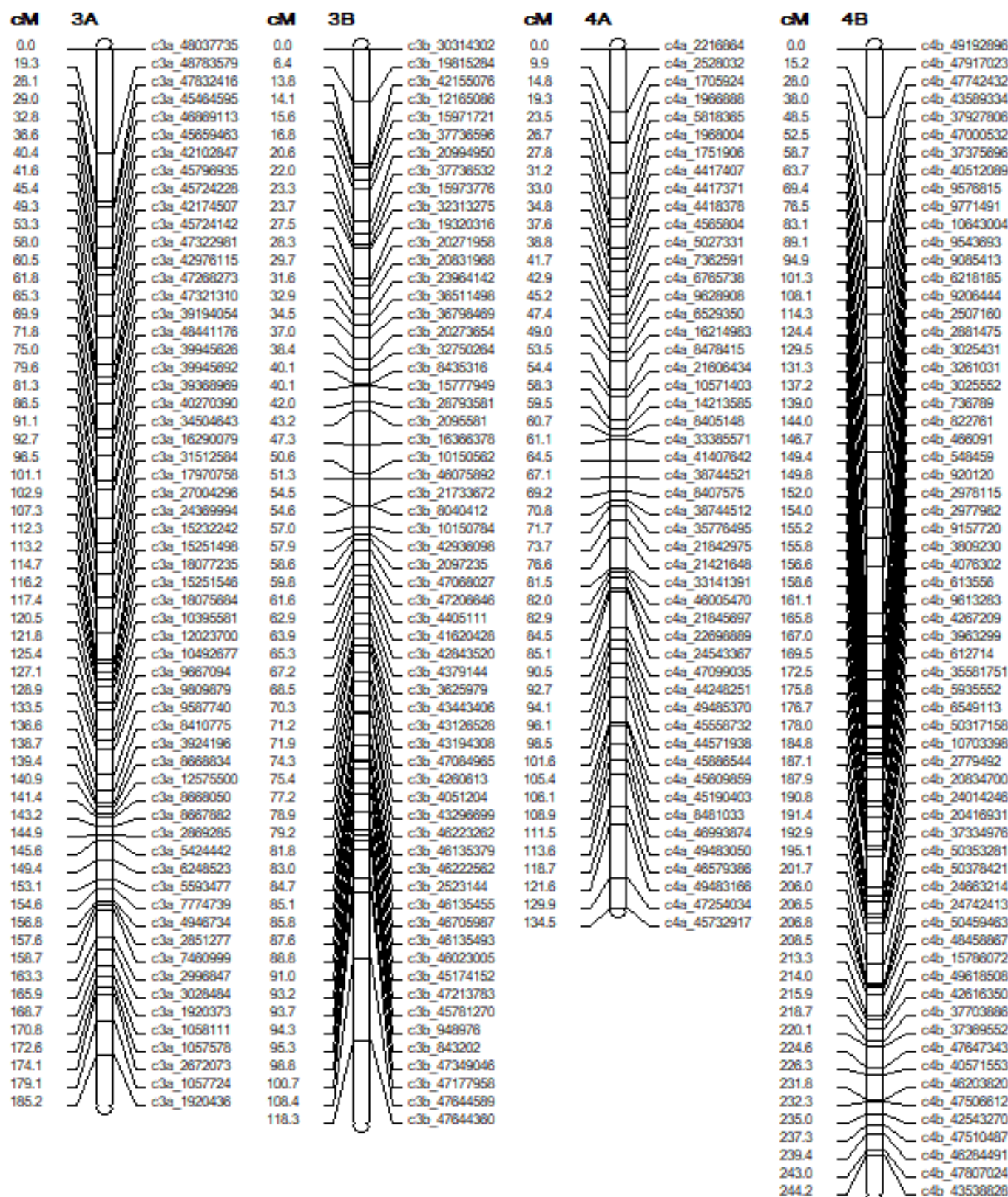

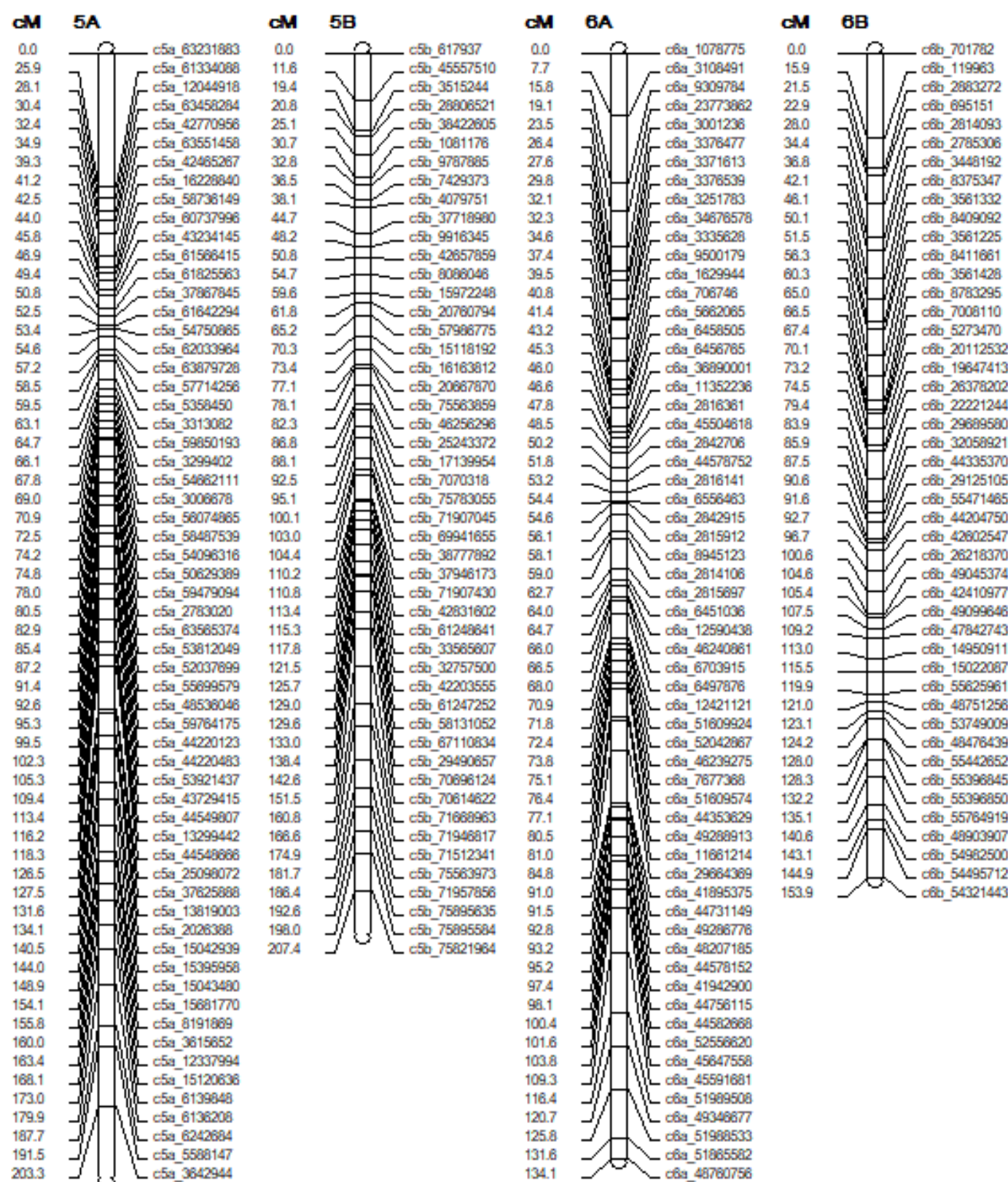

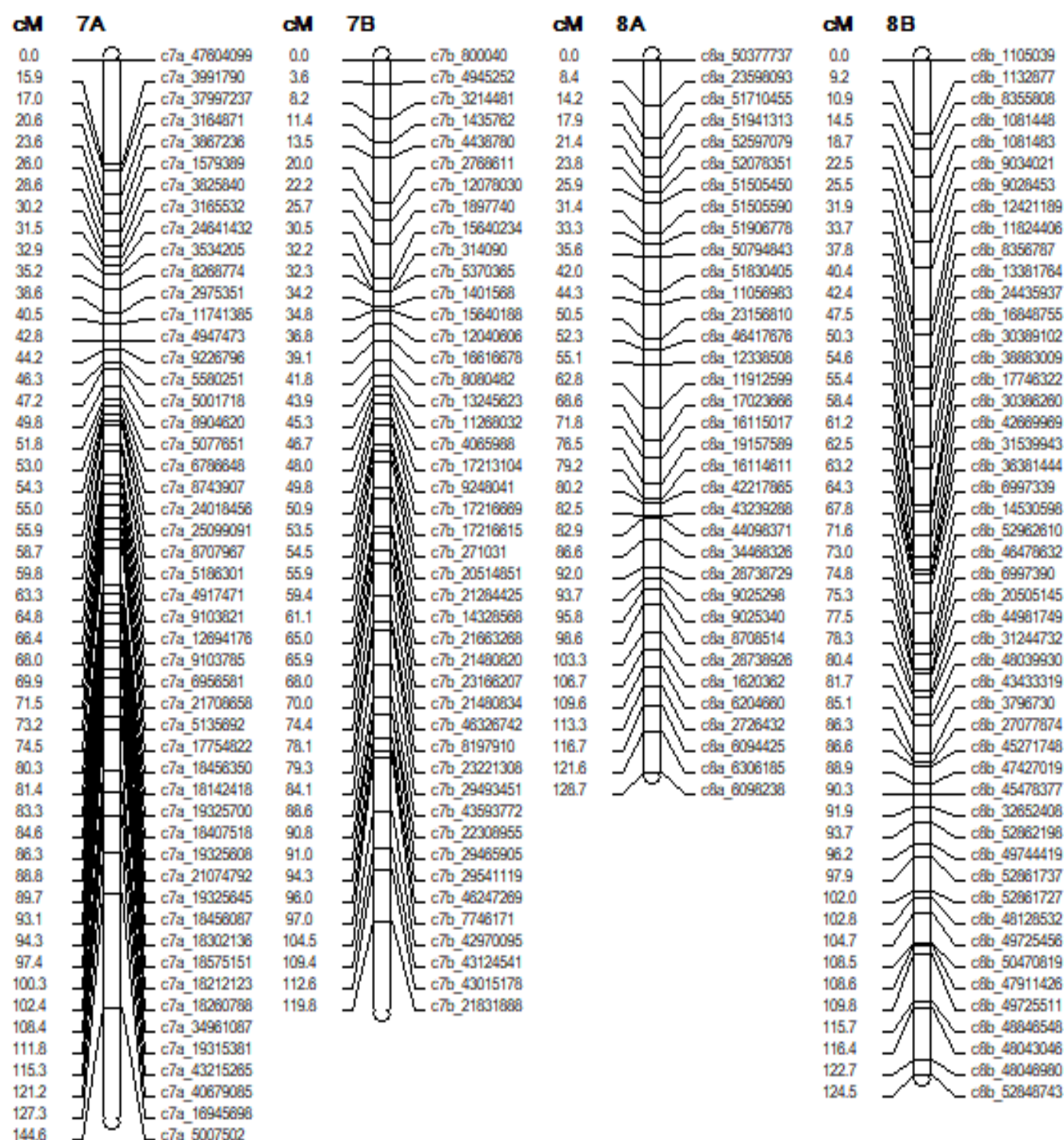

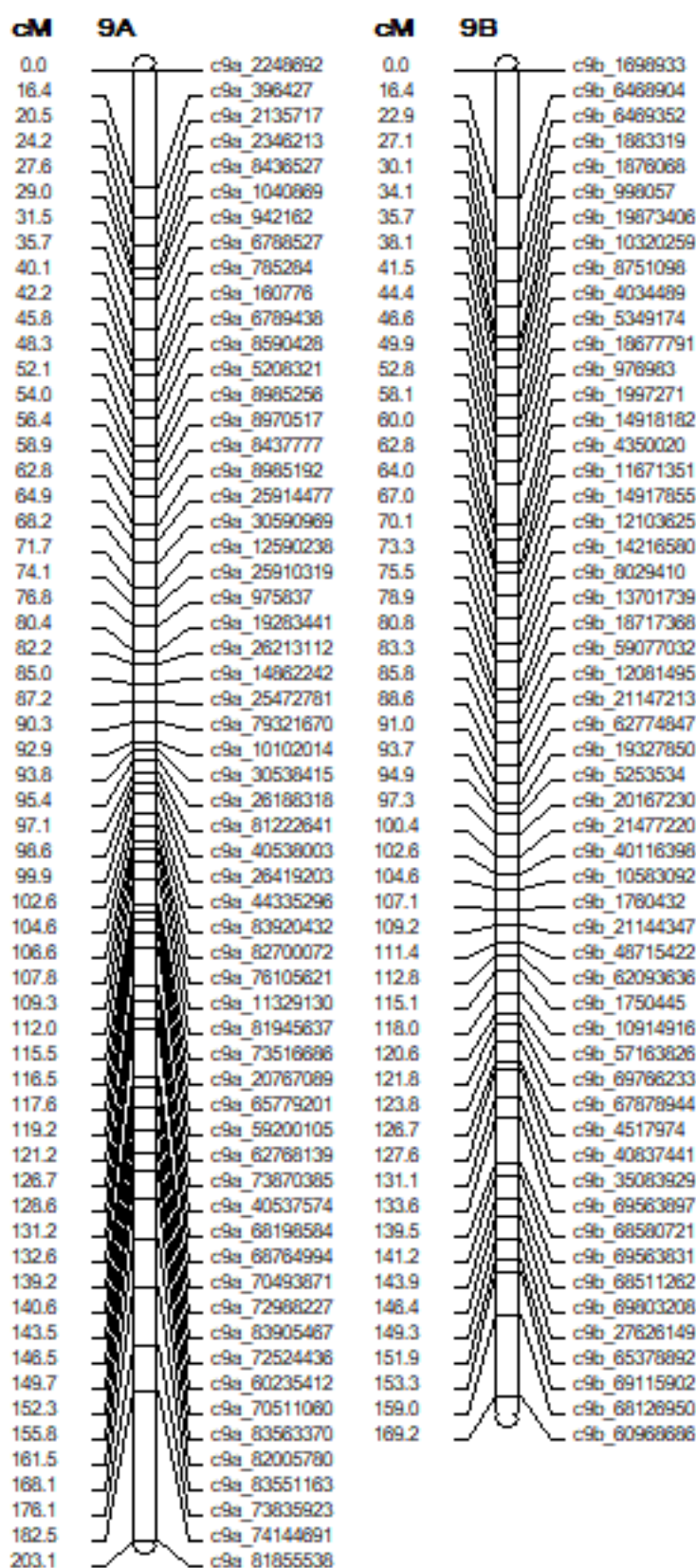

Supplement: Supplementary file 1 [file plants-11-00566-s001.zip › Figure S1.pdf]
